# Evaluating the effectiveness, efficiency, cost and value of contacting study authors in a systematic review: a case study and worked example

**DOI:** 10.1186/s12874-019-0685-0

**Published:** 2019-03-05

**Authors:** Chris Cooper, Juan Talens Bou, Jo Varley-Campbell

**Affiliations:** 1 University of Exeter Medical School, PenTAG, Exeter, UK; 2 Department of Clinical, Educational and Health Psychology, University College London, London, UK

**Keywords:** Supplementary searching, Author contact, Expert contact, Systematic reviews, Information retrieval

## Abstract

**Background:**

Studies find that identifying additional study data is possible by contacting study authors or experts. What is less certain is the time taken, costs involved and value found by using this supplementary search method.

The purpose of this study is to determine the effectiveness, efficiency, cost and value of contacting study authors by e-mail, updating the evidence available for this search method.

**Methods:**

Eighty-eight study authors, whose studies met title/abstract inclusion in a.

systematic review, were contacted by e-mail.

* effectiveness was assessed by comparing the number of study authors contacted.

compared to the number of replies received;

* efficiency was assessed by recording the time taken to contact study authors;

* cost was assessed by comparing the efficiency of contacting authors with the.

effectiveness; and

* value was assessed by reading and comparing the published studies with the replies received to see if any unique data was identified.

**Results:**

Contacting study authors took 6 h, 54 min and 25 s across 7 weeks. 38 answers (46%) were received from 83 possible contacts. Contacting study authors cost £80.33 or £2.11 per reply. We identified unique data from author replies when compared with data reported in published studies, determining this method as ‘valuable’.

**Conclusions:**

Whilst our effectiveness findings differ from other studies, we believe that this study demonstrates the effectiveness of contacting study authors. By linking effectiveness to value and cost, we offer a new way to interpret the ‘effectiveness’ of this supplementary search method.

**Electronic supplementary material:**

The online version of this article (10.1186/s12874-019-0685-0) contains supplementary material, which is available to authorized users.

## Background

Various studies have evaluated the effectiveness of contacting study authors or experts to identify unpublished studies or study data [1–6]. These studies find that identifying additional studies or study data is possible by contacting study authors or experts [1, 3–6], and that e-mail was an effective method of contact [2], being more likely to receive a reply than a letter [1]. What is less certain is the time it takes from identifying the need to contact authors and receiving replies, the costs involved, and the value found in the study data that this search method generates, if it is successful [7]. A recent review identified six studies [1–6] which evaluate the effectiveness of contacting study authors [8]. The publication dates of these studies range from 1989 to 2014, with the majority of studies being published before 2007 (*n* = 5/6). Only one study reported the time taken between contacting an author and reply (Gibson et al. (2006) and no studies reported data on costs. Given the advances in technology since publication of these studies, and the age of these studies more generally, we feel it is timely to update the evidence on this search method and we believe that we contribute data uniquely on the costs involved.

We contacted study authors as part of a systematic review [9]. We asked three questions of 88 study authors whose studies were included at full-text in our systematic review:

Question one: in your study, what did you categorise as effective or what was your measure of search effectiveness?

Question two: can you report any advantages or disadvantages you experienced in using the method in your study/studies, to evaluate literature search effectiveness?

Question three: in literature searching, what does effective (or effectiveness in) literature searching mean to you?

## Objectives

The purpose of this study is extending and updating the work of previous studies who have evaluated the effectiveness of contacting study authors [1]. The objectives of this study are:

1) to determine the effectiveness of contacting study authors by e-mail;

2) to determine the efficiency of contacting study authors by e-mail;

3) to determine the cost of contacting study authors by e-mail; and.

4) to determine the value of contacting study authors by e-mail.

## Methods

### Contacting authors

**Fig. 1:**
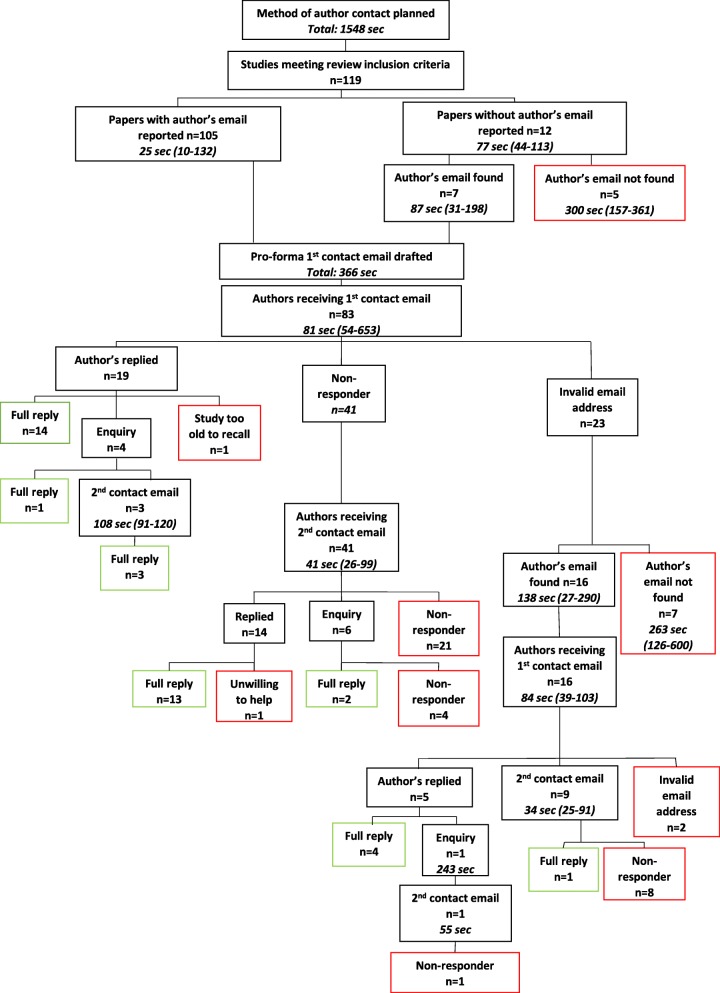
Schematic of contacting authors Key: *n* number, *sec* seconds

A data management plan was drafted in Excel 2013 by JTB, containing our agreed process for contacting study authors, and recording replies (Fig. 1). From the systematic review, 119 papers met full-text inclusion and these authors were eligible for contact [9].

A pro-forma e-mail was drafted, which sought answers to specific questions [10], and this was sent to the corresponding authors of each paper (see item one, Additional file 1). This e-mail was sent from an institutional e-mail account (@exeter.ac.uk) since O’Leary (2003) found that a ‘work based’ e-mail account may be ‘more valid’ as a form of contact compared to a Hotmail account [2]. The e-mail included University of Exeter branding and the names and signatures of the authors of this study and it is included as a supplementary file.

The e-mail address for the corresponding authors were taken from the studies included in our review. In the cases where no e-mail address was provided, we searched Google in an attempt to find a working e-mail address. If the corresponding author was not clear, we contacted the first study author. If an e-mail was returned with a delivery failure e-mail, the author’s details were searched using Google. If we could not locate a working e-mail address we recorded the study as ‘author’s e-mail not found’. If an author had more than one included study within the systematic review, they were only contacted once, about all their studies.

We allowed one month from sending the initial contact e-mail to accepting that author contact had failed. One month was chosen as a time-frame based upon the findings of Gibson et al. who reported an average response rate for author contact (generally) of 14 ± 22 days (median = 6 days) and e-mail (specifically) of 3 ± 3 days; median = 1. One month allowed for any variation in response rates for our work. After 14 days (two weeks), and if we had not received a reply from our initial e-mail, a reminder e-mail was sent from the same e-mail account as the first e-mail (see item two,Additional file 1).

### Determining effectiveness, efficiency, cost and value

We report the methods used to determine the above outcomes below. For effectiveness, efficiency and value, we have calculated and reported values using the same metrics as other studies that have evaluated the effectiveness of contacting study authors [1]. This will allow us to compare our findings alongside the work of Gibson et al.

### Effectiveness

The effectiveness of contacting authors was assessed by comparing the number of study authors contacted compared to the number of replies received. We recorded effectiveness results as n of contacts and n of replies and calculated percentages for ease of reporting.

### Efficiency

The efficiency of contacting authors was assessed by recording the time taken to contact study authors (e.g. drafting of e-mails, identifying e-mail addresses in the paper or via Google, sending follow-up e-mails) compared to the number of replies received. Timings were recorded using the stopwatch function in a Samsung Galaxy J7. All timings were recorded as set out in Fig. 1. We recorded efficiency in hours, minutes and seconds.

### Cost

The cost of contacting study authors was assessed by comparing the efficiency of contacting authors with the effectiveness. JTB, a graduate trainee, undertook all author contact and we report costs as time taken. JTB’s hourly rate was £11.50 (UK sterling, 2017).

### Value

The value of contacting authors was assessed by reading and comparing the published studies with the replies received to see if any unique data - i.e. data not reported in the original study - was identified. If we identified data not reported in the study, and that would provide unique information which informed the development of the thematic analysis linked to this wider study, we determined this as valuable, since it was data which informed the synthesis but that we would not have identified from the original study in question.

## Results

The process for obtaining the results is summarised in Fig. 1. The 119 studies eligible for author contact were published between 1978 and 2016.

### Effectiveness and efficiency: Generating the data management plan

The data management plan took 25 min 48 s to draft. Of the 119 studies included at full text in the systematic review, 88 unique authors were identified (some authors published multiple included papers and five authors’ e-mail contact details were unobtainable).

### Effectiveness and efficiency: Identifying contact details

Identifying e-mail address for the 88 unique authors from the 119 papers took a total of 1 h, 11 min and 41 s; a median of 25 s (range 10–132 s) per study.

Where an author’s e-mail address was unobtainable from the paper (*n* = 12), a total of 14 min 45 s was spent searching on Google for an alternative e-mail address; a median of 77 s (range 44–113 s) and seven new e-mail addresses were found. The publication date range for papers that did not provide email addresses ranged from 1978 to 2000.

### Effectiveness and efficiency: Contacting study authors (first contact e-mail)

It took 6 min 6 s to draft the first contact e-mail. Amending the e-mail template per author contacted took a total of 2 h 20 min and 27 s; a median of 81 s (range 54–653 s). We amended the template to include the name of the author and the study or studies we were contacting the author about, so that e-mails were tailored to authors.

Of the 83 e-mails sent, 14 authors replied first time with answers (17%) taking between 0 and 7 days to reply, one author replied stating that their study was too old to recall (it was published in 1996), 23 e-mails were returned as undeliverable (28%) and 41 authors did not reply (49%). A further four authors replied with an enquiry about our study before providing answers (5%). Our replies to these enquires took a total of 10 min 30 s.

Of the 23 e-mails that were returned as undeliverable, 16 new e-mail addresses were identified and seven were unobtainable. Identifying contact details of study authors from Google took a total of 1 h, 49 min and 38 s; a median of 156 s, range 27–600 s. Of these 16, four authors replied first time with answers (5%, of overall *n* = 83), one author replied with an enquiry (1%), two e-mails were returned as undeliverable (2%) and nine authors did not reply (11%). A total of 4 min 58 s was spent replying to the one enquiry, and chasing the enquiry two weeks later (ultimately no reply was received). In summary, 22% of total replies were received from the first successful e-mail contact of these 23.

### Effectiveness and efficiency: Contacting study authors (second contact e-mail)

A reminder e-mail was sent to all authors from whom we had not received a reply (*n* = 50, 60% of overall n = 83) or any indication that the initial e-mail was undeliverable. These second contact e-mails took 50 min and 6 s; a median of 39 s (range 25–99) to send.

A further 14 authors (28%) replied with answers, one author declined to answer the questions due to work burden, and 30 authors did not reply (60%) and were categorised as non-responders. Six authors replied with enquires (12%), where a further 3 min 32 s were spent replying to these (median 71 s, range 42–123 s), from which a further two answers were received and four failed to reply. In summary, 32% of total replies were received from the second e-mail contact of 50 authors.

### Effectiveness, efficiency and cost: Contacting study authors: Summary

**Fig. 2:**
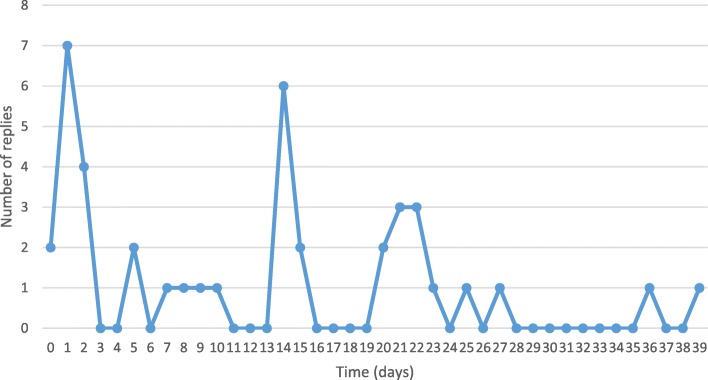
Number of responses received over time

In total, the process of contacting study authors took 6 h, 54 min and 25 s across 7 weeks. 38 answers (46%) were received from 83 possible contacts. Replies were received across 0–39 days (median 14 days) (Fig. 2). Contacting study authors in this study cost £80.33 in terms of total staff time or £2.11 per e-mail reply received.

In summary, from the initial intention of contacting 88 authors, we received 38 replies, 34 people did not reply (non-responders), we were unable to identify working emails for 14 people and 2 people replied saying they could not answer our questions.

### Value: Contacting study authors. Comparing the published studies with the replies received (Fig. 3)

#### Value: Question one

**Fig. 3:**
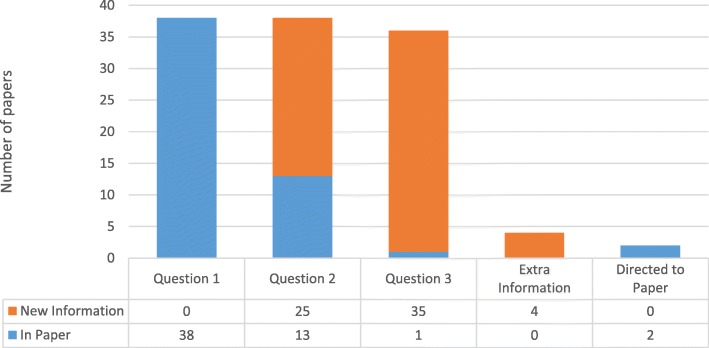
Value found in author replies

From the 38 authors who replied, 35 (92%) answered question one. One author answered question one four times (once for each of their included studies) therefore, 38 answers were received in total for question 1. Responses confirmed or clarified data reported in the studies (Fig. 3).

#### Value: Question two

From the 38 authors who replied, 35 (92%) answered question two. One author answered question two, four times (once for each of their included studies) therefore, 38 answers were received in total for question two. Twenty-five answers provided new information, whilst 13 did not (Fig. 3).

#### Value: Question three

From the 38 authors who replied with answers, 36 (95%) answered question three. Thirty-five answers provided new information, whilst one did not (Fig. 3).

#### Value: Additional information provided

Two authors did not reply to the questions but stated the answers to our questions were available in their paper. Four authors provided additional thoughts surrounding our questions, in addition to answering the questions (Fig. 3).

## Discussion

In discussion, we contextualise the findings reported above alongside other studies that have evaluated the effectiveness of contacting study authors.

### Effectiveness

Our effectiveness results broadly support the results reported in other studies that have sought to evaluate the effectiveness of contacting authors [1, 2, 4–6, 11]: that is, contacting authors is an effective method to identifying unreported or unpublished data. Understanding what constitutes an effective result, and therefore determining if this search method is worthwhile is, however, unclear.

Contextualising our response rate of 43%, alongside other studies that reported effectiveness outcomes in the same way (O’Leary 73%, Reveiz 7.6%) demonstrates the variability of response rates between studies, and it raises a potentially troubling question as to what constitutes effectiveness in this context. Whilst contacting authors is effective, in the sense that some authors do reply, the range of response rates above (including ours) demonstrates that contacting authors offers no guarantee of reply [12], and missing data remains likely, even in spite of extensive effort [3]. An evaluation of techniques to increase response rates from authors may be valuable since the ‘effectiveness’ of this search method is entirely conditional upon a reply [13].

E-mail offers a convenient, efficient and cheap way to make contact [1] and, with the advent of journals reporting author e-mail addresses in publications, finding a point of contact and making contact is more efficient than before. It is perhaps worth noting that, for studies pre-2000, we (unsurprisingly) found that journals did not report e-mail addresses. We also found that the way journals reported (or displayed) e-mail addresses varied between journals. In many cases, the e-mail address is reported alongside the authors names. In other cases, the e-mail address is recorded at the end of the study or in a footnote. Consistency in reporting between journals would improve efficiency for researchers.

Recording effectiveness in purely quantitative terms, as a response rate, gives no indication as to the value of the replies received. Whilst response rate is intuitive, the value of the replies received is a better metric to understanding if contacting authors is worthwhile, especially since this search method seeks to clarify or identify data not reported in the published study. Understanding the value found in replies would help define what data is (or can be) identified by contacting authors and this will inform the purpose of contacting authors and when it is worthwhile, or not. Unpicking this idea of value is however best understood when considered alongside efficiency (how long does it take to contact authors and how quickly do they reply).

### Efficiency (time)

We are not aware of any other study that has recorded the researcher time needed to contact authors (as a process), so we cannot contextualise the data presented in this study. It is worth noting that the time taken to contact authors will vary depending on the IT skills and proficiency of the researcher making contact. Recording the time taken to undertake literature searching - and undertake individual literature search methods specifically - may be useful since it can help inform decisions on how much time to allocate to the process of literature searching as a guide, as well as permitting some elementary form of cost-effectiveness analysis for the search method. Data on the time taken to search could be of particular use in resource or time-limited reviews, or when deciding whether or not to undertake a specific literature search [13].

In terms of efficiency, our results differ from Gibson et al. (2006). The response rate for replies is set out in Fig. 3, which shows replies were received across 39 days, but our median of 14 days was far higher than Gibson et. al’s (2006) 1.2 days [1]. We asked more questions and across a wider range of studies than Gibson et al. and our contact e-mails were sent by our graduate trainee (JTB). Young and Hopewell (2011) indicate the number of ‘items’ requested did not affect the probability of response, and the use of a well-known signatory also had no significant effect on the likelihood of authors reply [14]. We do not, therefore, think that the number of questions posed, or the fact that our graduate trainee sent the requests, altered the effectiveness of replies. We believe that the different responses rates demonstrate the fragility of this supplementary search methods, highlighting that, whilst researchers can contact study authors, there is no guarantee of a reply.

Selph et al. (2014) found that a reason for not sending data (in their study) was the time needed to find or format data [3]. We have set out the timing involved from our point of view but we have not accounted for the time taken by respondent authors to address our questions. Future studies might account for or ask for this data since it might impact of the likelihood of a reply.

A limitation of our e-mail sent to the study authors, was that it did not include a due date for responses. Working to deadlines are typical for most academics. Had we included a due date for response, we may have found an increase in the response rate. It is worth noting, however, that we did send a reminder e-mail which may have alleviated the effects of this.

### Cost

Cost is reported in this study as a tentative marker since costs (in terms of time) will vary not only by institution, salary and currency, but also variables such as the availability of contact addresses reported in studies.

We reported cost since it helps to contextualise the idea of value in effectiveness evaluation. As there was no comparator to e-mail contact in this study, we are unable to determine cost-effectiveness. We are not aware of any other studies that reported costs of contacting study authors so we are unable to contextualise our costs with other studies, or indirectly evaluate the cost-effectiveness of contacting study authors.

### Value

The content of the authors replies will be submitted for publication elsewhere since the focus on this paper is purely on the effectiveness efficiency, cost and value of contacting study authors as a supplementary search method.

We determined ‘value’ on the basis of ‘finding’ new information not reported in the study. In determining ‘value’, question one confirmed or clarified data available in the studies. This ‘type’ of question is common in systematic reviews [15] and it allowed us to report findings with confidence in the systematic review of effectiveness [9].

Question two sought to determine the advantages and disadvantages of evaluating literature search effectiveness. The ‘value’ of contacting study authors was perceptibly clearest here since the author replies contextualised issues experienced in the analysis of effectiveness evaluation which were not reported in the studies themselves. This explains the high number of replies which we classified as providing ‘new information’. The data provided has been analysed using thematic analysis, which has allowed us to develop a novel understanding of the issues experienced in evaluating literature search effectiveness. This work is submitted for publication elsewhere.

Question three, whilst linked to the spirit of this study, extends the question of literature search evaluation into a new domain. The replies here are all classified as providing ‘value’ because the question asked was not necessarily a part of the studies identified, even though it underpinned the purpose of the studies themselves. We found these replies valuable, since they provide answers to the broader question of why evaluate literature search effectiveness, and what is it that researchers are trying to measure. This question highlights an advantage of contacting study authors that we have identified elsewhere. When contacting study authors, you are able to ask questions of experts and capture data from their experience, if they reply [16]. Ogilvie et al. (2005) found that contacting experts was the link to better reports of studies already identified which helps illustrate this idea [17].

Whilst we associate author replies in this study with providing value, demonstrating the value found through literature searching, and through the use of one search method specifically, is a novel idea. In a case study comparing supplementary search methods to bibliographic database searching, we determined the value of search methods by the data found and any corresponding change to the synthesis of qualitative studies [18]. Similar ideas have been explored in meta-analysis, where studies have been included or removed from meta-analysis to mimic the effect of missing studies in literature searching [19]. In both cases, it is possible to gain a sense of the value found in individual studies, measured by the change in point estimate or findings available for synthesis. We believe that this idea of measuring value could advance the measurement of effectiveness in literature searching, allowing researchers to move beyond measuring sensitivity and specificity, to better articulate the wider point of why we literature search and what we identify [9]. As it relates to the data we identified here as valuable, we found richer explanatory data, which helped contextualise the rationale for measuring literature search effectiveness, benefits and problems in generating effectiveness estimates and, more broadly, findings that start to address the wider question of what researchers should measure to determine effectiveness. We intend to publish the thematic analysis relating to this work elsewhere and in due course.

## Conclusions

In this study, we have attempted to report and link together the effectiveness, efficiency, cost and value of contacting experts as part of a systematic review to evaluate the effectiveness of measuring literature search effectiveness. We believe that linking effectiveness of literature search methods (i.e. does it work?) to the value found (i.e. was it worth it?) is a way to advance the understanding supplementary search methods [7].

We have reported our values in a similar way to other studies. We believe that this is important since it allows greater contextual evaluation of studies and it will permit (as the number of studies grow) generalisability of outcomes measured. If more studies report the same outcomes, and in the same way, researchers will – in time – be able to generate approximate estimates as to the time required and ‘value’ found of supplementary search methods.

Whilst our effectiveness findings differ from other studies, we believe that this study demonstrates the effectiveness of contacting study authors. Linking effectiveness to value allows us to demonstrate the value we found and this extends the research available on contacting study authors since we can demonstrate the benefit beyond simply saying that authors replied. We also set out the costs and timings of the process from identifying the need to contact authors and receiving replies.

## Additional file

Additional file 1: Item 1 and 2. Copy of first and second pro-forma email sent to authors. (DOCX 21 kb)

## Abbreviations

n: number
