# Supplementary material for: Evaluating the effectiveness, efficiency, cost and value of contacting study authors in a systematic review: a case study and worked example
Source: BMC Med Res Methodol. 2019 Mar 5;19:45. doi: 10.1186/s12874-019-0685-0 (PMC6399953; doi:10.1186/s12874-019-0685-0)
Supplement: Supplementary file 1 — Item 1 and 2. Copy of first and second pro-forma email sent to authors. (DOCX 21 kb) [file 12874_2019_685_MOESM1_ESM.docx]

## Item 1

Dear Dr XXXXX,

We are undertaking a review that seeks to evaluate methods to estimate the effectiveness of literature searches. We read the following study of which you are the corresponding author.

*INSERT CITATION HERE*

As part of our work, we hoped to ask you three questions, two linked to your study/studies and the other linked more generally to the evaluation of literature search effectiveness. The answers will be used to help us understand the issue of measuring literature search effectiveness and we hope to integrate the replies into our study for publication.

If you can help, we should be grateful.

Yours truly,

Mr Chris Cooper,  Mr Juan Talens-Bou  and Dr Jo Varley Campbell

Question one: in your study, what did you categorise as effective or what was your measure of search effectiveness?

Question two: can you report any advantages or disadvantages you experienced in using the method in your study/studies, to evaluate literature search effectiveness?

Question three: in literature searching, what does effective (or effectiveness in) literature searching mean to you?

**Juan Talens-Bou**
Graduate Trainee Information Specialist
NIHR CLAHRC South West Peninsula (PenCLAHRC)
Tel: 0139272**3460**
[www.exeter.ac.uk](http://www.exeter.ac.uk)
Room 3.09, ESMI, South Cloisters, University of Exeter – St Luke’s Campus, College Road, Exeter, Devon, EX1 1TE

# Item 2

Dear Dr XXXXX,

Pursuant to my e-mail enquiry below, I wondered if you have had any time to consider our questions.

Yours,

Mr Chris Cooper,  Mr Juan Talens-Bou  and Dr Jo Varley Campbell

**Juan Talens-Bou**
Graduate Trainee Information Specialist
NIHR CLAHRC South West Peninsula (PenCLAHRC)
Tel: 0139272**3460**
[www.exeter.ac.uk](http://www.exeter.ac.uk)
Room 3.09, ESMI, South Cloisters, University of Exeter – St Luke’s Campus, College Road, Exeter, Devon, EX1 1TE
